# Ambient and focal attention during complex problem-solving: preliminary evidence from real-world eye movement data

**DOI:** 10.3389/fpsyg.2024.1217106

**Published:** 2024-02-15

**Authors:** Yuxuan Guo, Sebastian Pannasch, Jens R. Helmert, Aleksandra Kaszowska

**Affiliations:** ^1^Institute of Psychology III, Engineering Psychology and Applied Cognitive Research, Technische Universität Dresden, Dresden, Germany; ^2^Department of Electronic Systems, Aalborg University, Aalborg, Denmark

**Keywords:** two visual systems, problem-solving, think aloud, real-world eye movements, ambient attention, focal attention, fixation duration, saccade amplitude

## Abstract

Time course analysis of eye movements during free exploration of real-world scenes often reveals an increase in fixation durations together with a decrease in saccade amplitudes, which has been explained within the two visual systems approach, i.e., a transition from ambient to focal. Short fixations and long saccades during early viewing periods are classified as ambient mode of vision, which is concerned with spatial orientation and is related to simple visual properties such as motion, contrast, and location. Longer fixations and shorter saccades during later viewing periods are classified as focal mode of vision, which is concentrated in the foveal projection and is capable of object identification and its semantic categorization. While these findings are mainly obtained in the context of image exploration, the present study endeavors to investigate whether the same pattern of interplay between ambient and focal visual attention is deployed when people work on complex real-world tasks—and if so, when? Based on a re-analysis of existing data that integrates concurrent think aloud and eye tracking protocols, the present study correlated participants’ internal thinking models to the parameters of their eye movements when they planned solutions to an open-ended design problem in a real-world setting. We hypothesize that switching between ambient and focal attentional processing is useful when solvers encounter difficulty compelling them to shift their conceptual direction to adjust the solution path. Individuals may prefer different attentional strategies for information-seeking behavior, such as ambient-to-focal or focal-to-ambient. The observed increase in fixation durations and decrease in saccade amplitudes during the periods around shifts in conceptual direction lends support to the postulation of the ambient-to-focal processing; however, focal-to-ambient processing is not evident. Furthermore, our data demonstrate that the beginning of a shift in conceptual direction is observable in eye movement behavior with a significant prolongation of fixation. Our findings add to the conclusions drawn from laboratory settings by providing preliminary evidence for ambient and focal processing characteristics in real-world problem-solving.

## 1 Introduction

### 1.1 Two visual systems reviewed

Vision is a sense that allows to sample the information from our surroundings, select and code different aspects of information, and produce visually guided behaviors to interact with the world (James, 1890). Understanding vision and visual perception is of long-lasting interest. Over the last decades, research into vision has proposed a model that made distinctions between the functional organization of the two visual processing systems.

In the late sixties, Trevarthen (1968) postulated an anatomical separation between vision of space and vision of objects based on experiments with split-brain monkeys, and named the two visual systems *ambient* and *focal*. The ambient system provides a visual spatial frame for action centered on the body as a whole. The focal system, on the other hand, functions as a high-resolution analyzer system centered around the foveal projection. At about the same time, Schneider (1967) who described experiments on golden hamsters with brain lesions also came to the conclusion that there were two visual systems: one responsible for the coding of location (‘where’), and one responsible for the identification of the stimulus (‘what’). These distinctions marked a significant departure from earlier descriptions of the model of two visual systems. The model of two visual systems has developed and gradually crystallized over years with experimental support from a variety of research fields. Later perspectives on modularity in the two visual systems placed more emphasis on output distinctions, e.g., visual control of action vs. visual perception. Based on behavioral evidence from lesion studies, Ungerleider and Mishkin (1982) distinguished two broad cortical pathways of visual processing, the dorsal and ventral streams. This proposal paved the way for the later model of visual processing which differentiates between vision for action and vision for perception (Goodale and Milner, 1992; Goodale et al., 1994; Milner and Goodale, 1995; Milner and Goodale, 2008). Milner and Goodale’s (1995) version of the model of two visual systems takes more account of the functional standpoint that separate visual pathways are specialized for different uses to which vision can be put. The dorsal stream transforms visual information about the goal object into the appropriate coordinates of surrounding arrays in real-time, providing a viewer-centered framework and playing a role in the guidance of our actions in picking it up. The ventral stream transforms visual inputs into rich and detailed perceptual representations that encode the size, orientation, and location of objects relative to the other, as well as its semantic properties, allowing us to parse the scene and to recognize objects (and their relations) in the visual world.

Even though the above-mentioned studies generated slightly different terms and definitions, the distinction between egocentric spatial frame and object recognition, between ‘ambient’ and ‘focal’, has persisted in visual neuroscience (Bridgeman, 1991; Andre and Rogers, 2006; Rooney et al., 2017; Owens et al., 2018). More importantly, this ambient-focal dichotomy has also been applied to the field of eye movement research. According to Trevarthen (1968), the course of interplay between the ambient and focal system, such as the ongoing perception of object identities within appropriate spatial context, also closely depends upon the oculomotor adaption. During normal daily activities, such as reading a newspaper or viewing an image, oculomotor activity can be described as an interplay between saccades and fixations (Yarbus, 1967). Saccades—fast ballistic movement of the eyes—direct the small high-resolution foveal region from one point of interest to another. The relatively stable periods between saccades are known as fixations, within which the intake of high-resolution visual information occurs. These two parameters of oculomotor behavior effectively change to favor one or other visual mode: a large field of vision is explored by larger saccades, which gives the impression of a strong stimulation of peripheral spatial apprehension, and therefore a fall in high-acuity focal attention. Foveal resolution of configuration, pattern, hue, etc., within the spatial context is organized during successive intent fixations, which gives the impression of stimulation of a high-acuity analyzer system and depresses peripheral ambient attention.

The concept of using conjugate eye movements as the most direct sign of the interplay between two visual systems has been sharpened by a series of eye movement studies of scene perception. In static scene viewing research, Antes (1974) was one of the first who reported an increase in fixation durations together with a decrease in saccade amplitudes over the time course of scene inspection. Unema et al. (2005) further investigated the systematics in information processing during static scene viewing, demonstrating changes in fixation durations and saccade amplitudes over time. The authors interpreted the observed fixation and saccade behavior with the ambient/focal visual systems approach (Trevarthen, 1968). Specifically, in Unema et al. (2005), the short fixation durations and long saccade amplitudes observed during the early viewing periods were interpreted as ambient mode of vision, which may be used to structure a spatial frame and determine the whereabouts of the objects in the scene. Having the spatial frame stabilized makes it possible to pay closer attention to an object of interest and analyze it in detail. Correspondingly, the longer fixation durations and shorter saccade amplitudes observed during the later viewing periods were interpreted as focal mode of vision. This interpretation is supported by Velichkovsky et al. (2005), who found that objects/regions of a scene fixated in the course of focal processing (when fixation duration was long and the subsequent saccade amplitude was short) were better recognized than those similarly fixated in the course of ambient exploration (when fixation duration was short and the subsequent saccade amplitude was long) in a recognition task. Further evidence for transitions between ambient and focal visual processing in static scene viewing was provided by Pannasch et al. (2008) who studied the time course of eye movements while participants viewed scenes under various conditions. Their data demonstrated a systematic increase in fixation durations together with decreased saccade amplitudes over a period of 6 s across different natural viewing conditions. This default-like viewing behavior was repeated after the presentation of a new stimulus, suggesting that ambient to focal processing was evoked by the onset of a new visual environment and served as basic attentional mechanism in static scene viewing independently of viewing condition.

Comparable research into eye movements during more naturalistic dynamic scene viewing demonstrates the same dichotomy between ambient and focal eye movement characteristics. First indications of a dynamic balance between the ambient and focal processing were reported by Velichkovsky et al. (2002) analyzing hazard perception during a driving simulation task. Later, Smith and Mital (2013) compared viewing gaze behavior of dynamic to static scenes and found that dynamic scene viewing exhibited similar eye movements to previously described static scene viewing. Finally, Eisenberg and Zacks (2016) investigated patterns of eye movements while participants viewed naturalistic movies or live action. They found that participants shifted from focal to ambient processing (decreasing fixation durations and increasing saccade amplitudes) around subjective event boundaries between meaningful units of activity. The authors suggested that event boundaries function similarly to the onset of a new scene during static scene viewing. Because people need to update their working memory representations when features of the current environment change and activity becomes less predictable, this event model update entails ambient eye movements for exploratory processing around event boundaries.

The interplay between ambient and focal visual processing is not only identified in relation to external events in the visual environment, such as the onset of new stimuli or subjective event boundaries as discussed above. First evidence suggests that the interplay between these two modes of processing also takes place during the performance of more complex tasks without any external changes. In a recent study by Guo et al. (2022), participants engaged in an extended task that required them to switch between different sub-tasks until the macro task was completed. After participants switched from one sub-task to another, the authors found indications of ambient to focal visual processing shift (increasing fixation durations and decreasing saccade amplitudes) over the processing time between different sub-tasks. Extending previous research, Guo et al. (2022) suggested that the shift between ambient and focal attentional processing can be triggered internally, closely related to the inner mental model, such as mental shifts between different tasks.

Taken together, characteristics in eye movement patterns adequately reflect the functional dichotomy and the transition between the ambient and focal visual system. Short fixations and long saccades are optimal for “driving” ambient vision extending over the whole visual field, which is of the ability to adapt to spatial alterations or to extensive temporo-spatial relationships—for example, stimuli onset/activity changes in scene viewing tasks trigger ambient gaze behavior as demonstrated in (Unema et al., 2005; Pannasch et al., 2008; Eisenberg and Zacks, 2016), or the mental shifts between different tasks in (Guo et al., 2022). In contrast, longer fixations and shorter saccades are essential in maintaining the resolving power of focal vision, which is of decisive importance to the recognition and learning of complex details.

### 1.2 Visual attention deployment in complex problem-solving

Although the ambient and focal visual processing phenomenon has been replicated in numerous studies, it has yet to be investigated in more naturalistic settings. The use of naturalistic stimuli within laboratory studies, e.g., the real-world scenes utilized in scene viewing tasks (see Pannasch et al., 2008; Smith and Mital, 2013), provides a bridge between laboratory and naturalistic settings. However, a deeper understanding of visual processing mechanisms cannot be achieved without determining whether the results derived from images depicting real-world scenes generalize to the visual world itself, that is, to the situation in which the participant is looking at and interacting with the real-world dynamic environment. To this effect, a more natural and complex task, such as solving problems in real-world settings that requires participants to continuously interact with the environment, would be appropriate. Prior research has demonstrated a strong relationship between attention deployment and problem-solving by showing the activation of attention-related brain areas as people solve problems (Kounios et al., 2008; Kounios and Beeman, 2009). More importantly, eye tracking studies extended prior research by providing direct evidence for a relationship between visual attention and problem-solving, e.g., eye movements and blinks have revealed dynamic differences in overt attention when people solve problems with sudden insight versus analytically (Salvi et al., 2015).

However, the deployment of visual attention in problem-solving was mostly investigated in laboratory settings as people solved short, highly structured, visually presented problems (e.g., Knoblich et al., 2001; Rehder and Hoffman, 2005). Compared to laboratory tasks, problems in real life are often more fuzzy, ill-structed, and require more voluntary control of visual attention for gathering and refining relevant information contained within the environment (Simon, 1978). Ill-structed problems are defined by having an ambiguous problem state, incomplete or ambiguous goals, which do not include only one specific solution path. To solve ill-structured problems, solvers need to integrate multiple knowledge domains and to constantly interact with the environment in order to systematically evaluate possible problem states intervening between the starting state and the goal state (Newell and Simon, 1972; Kounios et al., 2008). The complexity of such problems can be managed through decomposition of the problem into a series of smaller sub-problems that are easier to address in sequential steps. Correspondingly, in ongoing problem-solving, solvers are likely to encounter difficulties when working on possible solution paths through steps, which results in impasses, and forces solvers to adjust their conceptual direction. To overcome an impasse, the solver must address and reevaluate the troubling issue, shift between possible ideas or approaches to define or update the problem state (a reorganization of the conceptual representation of a problem), eventually conclude with a solution (Ohlsson, 1992; Ollinger et al., 2014).

The important role of perceptual processes in problem-solving, especially in the generation of the solver’s internal representation of the problem, was demonstrated early by Simon (1978) as well as Newell and Simon (1972) in their ‘information-processing theory of human problem-solving’. In particular, their theory postulates that the framework for problem-solving behavior is established by three components: information-processing system, task environment, and problem representation. The information-processing system is an adaptive system that relates problem representation and task environment to each other. Solving a problem may require drawing upon large stores of information in long-term memory and in external reference sources, e.g., the task environment. The task environment refers to an environment containing a goal, a problem or task, as well as the amount of semantic information necessary to solve the problem. As outlined above, solving a problem is an odyssey through different problem states—what the solver knows about the problem at a particular moment of time—until the current state of knowledge includes the solution to attain the goal. To set forth from one problem state to another, in other words, updating the state of knowledge, the solver must gather or retrieve relevant information available in the task environment and successfully embed critical features into his conceptual representation of the problem (Kaplan and Simon, 1990; Ollinger et al., 2014, 2017).

Given that problem-solving behavior can be characterized as a series of elementary information processes organized into strategies or programs (Simon, 1978), when the task environment remains constant, a deployment of attentional strategies for information-seeking behavior may be necessary to sharpen the problem-solving efforts. This leads to our research question: What role does the interplay between ambient and focal visual attention play when people are working on a realistic complex problem? As previously mentioned, ambient/focal visual attention is governed by neural processes that have been argued to support spatial representations or object-oriented (detail-based) representations. Ambient and focal attention are likely involved in just that—the representation of said information, with other processes/mechanisms using that information to intelligently guide thought and behavior. The processing of information operates through one or other visual attention inherently driven by the goals of the observer. To gain further insight into our research question, we conducted a reanalysis of data from an eye tracking study (Kaszowska, 2019). This study utilized a tool design task to explore complex problem-solving in a real-world setting and understand how individuals adjust their conceptual direction when working on a complex problem. The chosen complex ill-structured tool design task served as a testbed for investigating problem-solving strategies and complex cognition, with a specific focus on examining the link between attention, gaze deployment, and verbalization in naturalistic scenarios.

**Figure 1:**
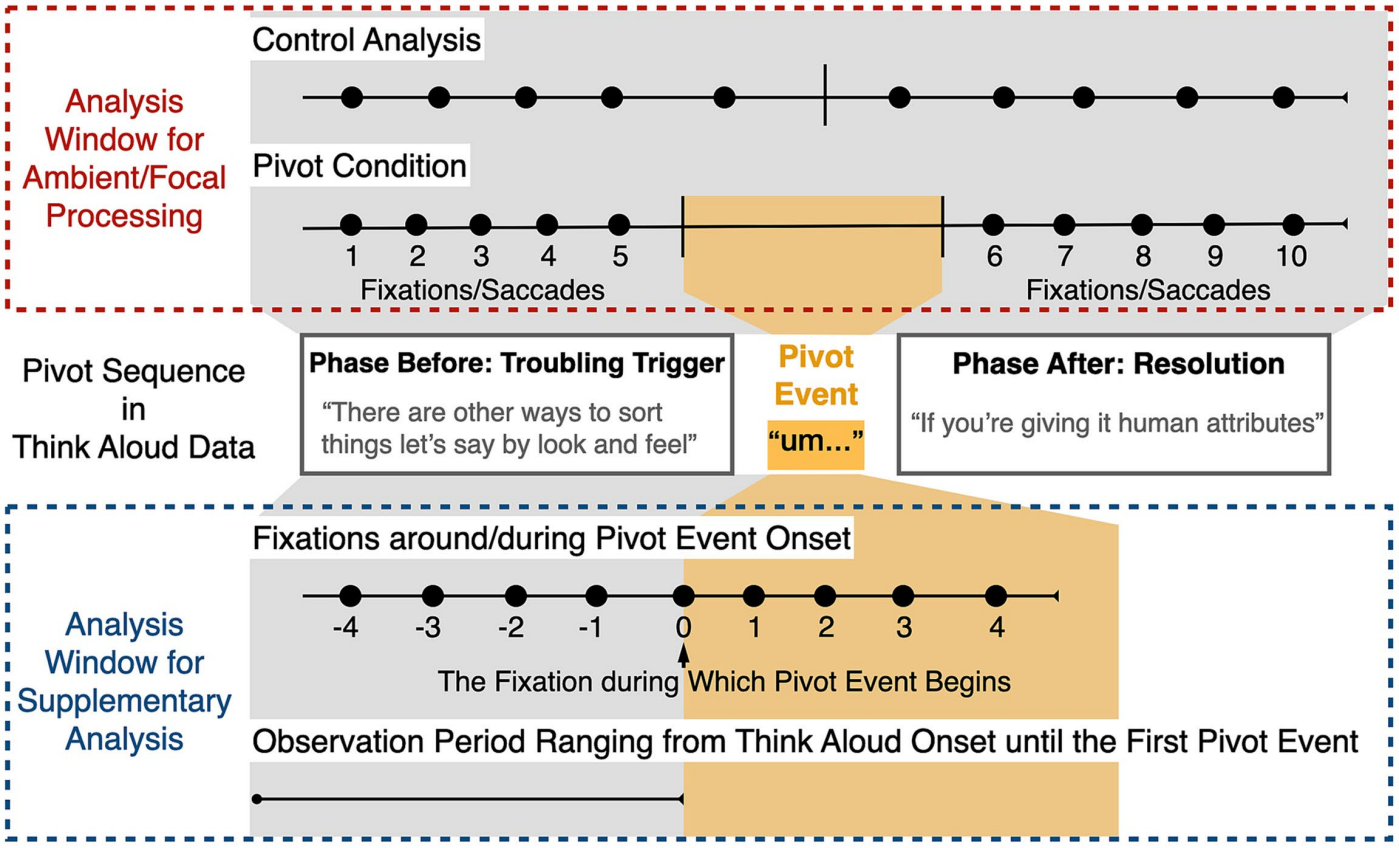
Diagram depicting the analysis windows. The pivot sequence in think aloud data is presented in accordance with its stages. The red frame illustrates the numbering of a sequence of 10 fixations/saccades selected for the pivot and the control analysis for testing whether transitions between ambient and focal processing occur in tandem with pivot events. The blue frame illustrates the numbering of a sequence of 9 fixations selected for the investigation of visual fixation responses to the onset of a pivot event, as well as the defined observation period for evaluating fixation durations before any pivot event.

In Kaszowska (2019), 45 engineering students had to conceptualize a tool—a physical piece of equipment—that could be used by a specific end user (either a robot, a human, or a team consisting of both robot and human) in sorting Lego blocks into individual containers according to a reference image depicting a sorted kit. The purpose of this prospective tool was to facilitate and optimize the sorting process. This tool design task only specified the initial state (a need for a tool) and the end state (a tool improves sorting efficiency) of the problem, without predetermining any solution path. Participants planned possible solutions for approximately 10 min while thinking aloud (i.e., participants were verbalizing their ongoing thought processes). The concurrent think aloud reflects ongoing cognitive activities, such as what information is being processed and how it is processed (Ericsson and Simon, 1980; Russo et al., 1989; Payne, 1994). During this planning process, participants could manipulate the Lego pieces, refer to both the reference image and the end user’s appearance. While free-viewing information contained within the environment, their eye movements were recorded using a mobile eye-tracker. Kaszowska (2019) combined the concurrent think aloud with eye tracking recording to analyze how participants engaged with ideas when exploring possible solution paths to the tool design problem. In particular, the author content-coded the think aloud data and isolated specific instances where the verbalization content indicated a shift in conceptual direction and termed them *pivots* and *pivot sequences*. A pivot sequence is initiated during the pivot when the need for updating or redefining the problem representation arises. More precisely, the pivot sequence is defined as a three-stage problem-solving process, which starts with encountering a troubling issue, responding with a pivot (recognizing an issue), and then resolving the issue immediately. Given the nature of concurrent verbalizations, which reflect how designers engage with design ideas and what cognitive processes contribute to the ongoing planning and problem-solving, a thorough analysis of speech content units can effectively capture moments of hesitation, uncertainty, or the evaluation of small decisions and judgments. Functionally, hesitation markers in language production have been theorized to create time for verbal planning in speech (Maclay and Osgood, 1959; Rochester, 1973). In many cases, hesitations are relevant for communication and provide information about underlying cognitive processes (Brennan and Williams, 1995; Tenbrink et al., 2011). As such, the pivot is often reflected as a hesitation marker in participant’s verbalizations, which is essentially a verbal event (including filled nonverbal content such as “ums” and “uhs” or silences); refer to Figure 1 for an example of a pivot sequence in think aloud data, illustrating how the content units of verbalization change over the three stages—considering alternatives, pivot, and proposing a design idea.

To explore perceptual and attentional processes in how humans interact with the surrounding world, Kaszowska (2019) introduced two categorical predictors to model oculomotor behavior: pivot kind (denoting whether the conceptual shift occurred within an idea or between ideas) and fixation kind (categorize eye gaze on regions of interest with respect to verbalization content). This approach yielded valuable insights into the intricate relationship between visual attention and speech. Specifically, the author explored how guiding *look-ahead* fixations (i.e., fixations anticipating interactions with the subsequent pivot resolution phrase content) and monitoring *look-back* fixations (i.e., fixations monitoring interactions with the preceding troubling trigger phrase content) accompany changes in conceptual direction (reflected as pivot sequences in concurrent think aloud data). Look-ahead and look-back eye movements are extensively studied in the literature in the context of active vision, specifically in the interplay between vision and movement such as reaching and grasping. Look-ahead fixations are prominent during natural tasks, where participants sometimes fixate on objects before manipulating them (Pelz and Canosa, 2001; Mennie et al., 2007), suggesting that guiding eye movements are purposeful and have a role in task planning. In contrast, look-back fixations return to a previously inspected region of interest within a narrow temporal window after an action has been completed (Gilchrist and Harvey, 2000; Mennie et al., 2007). The study (Kaszowska, 2019) unveiled a tight linkage between eye movements and verbalization during problem-solving, demonstrating that the guiding and monitoring processes were differentially deployed over the course of conceptual direction shifts. In pivot sequences, where participants are more likely to evaluate various independent ideas or approaches, look-ahead fixation were more frequent 1 s before and after the pivot compared to during the pivot (i.e., the resolution phrase content is more frequently attended to before and after the given pivot). On the other hand, during smaller-scale conceptual shifts—pivot sequences where participants are more likely to alter between various aspects of the same idea—the proportion of look-ahead fixation (the focus on resolution phrase) decreases over the pivot sequence. The frequency of monitoring look-back fixation remains stable throughout the pivot sequence, irrespective of the pivot magnitude (e.g., a pivot either denotes a shift to a different idea or a shift to a different aspect of the same idea), implying that the involvement of monitoring processes is invariant of conceptual shift scope. Additionally, the study reported a variation in fixation duration as a function of pivot magnitude (pivots denote a shift to a different idea are accompanied by shorter fixations) and pivot sequence stage (fixation durations increase over the course of a pivot sequence).

This paradigm (Kaszowska, 2019) is methodologically advantageous for addressing our research question because (a) the objects in the task environment (e.g., the end user and the reference image) are rich in semantic information that must be supplied to solve the tool design problem, which requires a role for perceptual processes in the given context. (b) Recording eye movements and verbal think aloud protocol effectively provide a high temporal density of observations for exploring the interaction between perceptual and cognitive processes in problem-solving. The deployment and interplay of ambient and focal attention for visual information processing will be reflected in moment-by-moment changes in the characteristics of eye movement patterns throughout the course of the given problem-solving task.

### 1.3 Ambient-focal attentional strategies in problem-solving

In light of previous findings, we formulate our hypothesis with the underlying assumption that the interplay between ambient and focal processing mechanisms is discernible in relation to changes in inner mental models, such as when people update their event model of the current environment (Eisenberg and Zacks, 2016) or when they switch between different tasks (Guo et al., 2022). Therefore, one possibility is that a shift in conceptual direction during problem-solving might entail a shift between ambient and focal visual attention. Because shifts in conceptual direction typically occur when people identify a troubling issue, and when their conceptual representation of the problem is incomplete or incapable of leading to the solution. To resolve the troubling issue, a qualitative attentional shift would be a reasonable strategy to facilitate the restructuring and updating of the problem representation during this timeframe. However, the precise nature of such visual attention shifts remains ambiguous, given that the processing of information occurs through different attentional strategies depending largely on the current viewing goals of the designer. In Kaszowska (2019), when designers change their conceptual direction, the troubling trigger and resolution phrases within a pivot sequence may transition either within the same content category (reflecting sustained focus on a particular object or event) or between different categories (reflecting a shift in general focus). More importantly, in the given task, eye movements play both the guiding and monitoring role in active vision (referred to as look-ahead and look-back fixations, as previously discussed), and these guiding and monitoring eye movements are differentially involved in various conceptual direction shifts, demonstrating that participants were involved in different ways of interacting with information. As such, the attentional shift strategy facilitating conceptual direction shifts could vary from trial to trial. For instance, it is likely that people engage in ambient exploratory processing at the troubling trigger’s onset—in the form of relatively short fixation durations together with long saccade amplitudes—in order to gather broad information within the egocentric spatial framework (task environment) and register features or events that might be associated with the troubling issue. Whereas after a shift in conceptual direction, a determined solution pops into mind, information about the space within the ambient field provides context for the ordering of focal perception within a specific part of the field—in the form of longer fixation durations together with shorter saccade amplitudes—which enable people to filter out distracting or irrelevant information and to think about the solution content represented in the conceptual system. On the other hand, the other direction—a transition from focal to ambient processing—seems also feasible. Suppose that a shift in conceptual direction represents the moment when the solver is heavily focused and realizes that he has reached an impasse, compelling him to explore new ideas. It is therefore possible that people maintain focal processing of the current problem representation until the realization of an impasse that demands a shift in conceptual direction. Once the conceptual direction has shifted, people switch from focal to ambient processing in order to capture as much information as possible for refining the mental representation of the newly concluded solution. Considering that individual characteristics, such as prior or advanced knowledge (Gralla, 2014), age (Griffin and Spieler, 2006), expertise (Wineburg, 1991), or cultural background (Kim, 2002), influencing how speakers perceive situations and approach tasks also make a difference in verbalizations, individuals may exhibit preferences for different attentional strategies, such as ambient-to-focal or focal-to-ambient.

Preliminary evidence for the temporal change in eye movement behavior around pivot events was reported in Kaszowska (2019); fixation durations showed a significant increase over the pivot sequence stage (before, during, and after a pivot). Nevertheless, the original study did not provide a time course of fixation durations and saccade amplitudes throughout the periods preceding and following pivot events, nor a comparable control analysis which could lead to an evaluated probability of having distinct eye movement patterns due to the presence or absence of pivot events.

Our analysis of the data by Kaszowska (2019) therefore sought to examine temporal changes in fixation durations and saccade amplitudes during the periods around conceptual direction shifts (reflected as pivot events in think aloud data). For simplicity, below we describe the conceptual direction shift as “pivot event” according to Kaszowska (2019). Specifically, we hypothesized that pivot events would be accompanied by transitions between ambient and focal visual processing. If participants employed the ambient-to-focal attentional strategy, fixation durations would be short and saccade amplitudes would be long during the periods when they were approaching the pivot events. In contrast, in the periods following pivot events, fixation durations would become longer and saccade amplitudes would become shorter. Alternatively, if participants employed the focal-to-ambient attentional strategy, eye movement behavior would exhibit reversed temporal changes (decreased fixation durations and increased saccade amplitudes) during the periods around pivot events. However, such characteristics should not exist in a condition without pivot event. In a comparable control analysis (without any pivot event), where participants were fluently verbalizing the content of their thoughts without altering the conceptual direction, fixation durations and saccade amplitudes would be maintained at a steady level. It is also worth emphasizing that not every shift in conceptual direction necessarily coincides with a transition between ambient and focal visual processing. In other words, individuals may not consistently alter their gaze behavior in response to pivot events. Throughout a self-paced problem-solving process, it is likely that an individual exhibits varying patterns of gaze behavior during the periods around pivot events, including cases where gaze remains unchanged, transitions from ambient to focal processing, or transitions from focal to ambient processing. Nevertheless, our primary focus here is to investigate the predominant visual processing mechanism employed in the context of complex problem-solving. Consequently, we are interested in detecting qualitative attentional shifts in complex problem-solving as well as in a better understanding of the correspondence between parameters of eye movement and the temporal dominance of one of the two modes of visual processing.

## 2 Materials and methods

For our analysis, we used the data set from a real-world tool design task as an approximation to a real-life problem-solving situation. Below, we present an overview of the experiment setup, the data, and the methodology upon which we built our current work.

### 2.1 Tool design task

#### 2.1.1 Ethics declaration

The study was approved by Tufts University Social, Behavioral, and Educational IRB under protocol approval number 1502022 (Kaszowska, 2019).

#### 2.1.2 Participant information

Forty-five participants (23 women, all native or near-native English proficiency) undergraduate and pre-master’s students (age *M* = 19.7, *SD* = 2.5) from Tufts University completed the study for monetary compensation. Participants were randomly assigned to one of three different conditions, each confronting a different intended end user for the tool (robot; human; or a team consisting of robot and human). Fifteen participants were randomly assigned (8 women in robot and human scenarios, respectively; 7 women in team scenario) to the conditions. Participants came from engineering (mechanical, civil, biomedical, environmental, electrical, and general), human factors, and computer science study programs.

#### 2.1.3 Recording

Eye movement and think aloud recordings were performed using SMI Mobile Eye Tracking Glasses (ETG-1; SensoMotoric Instruments, Inc., Germany), at 30 Hz.

#### 2.1.4 Task materials

**Figure 2:**
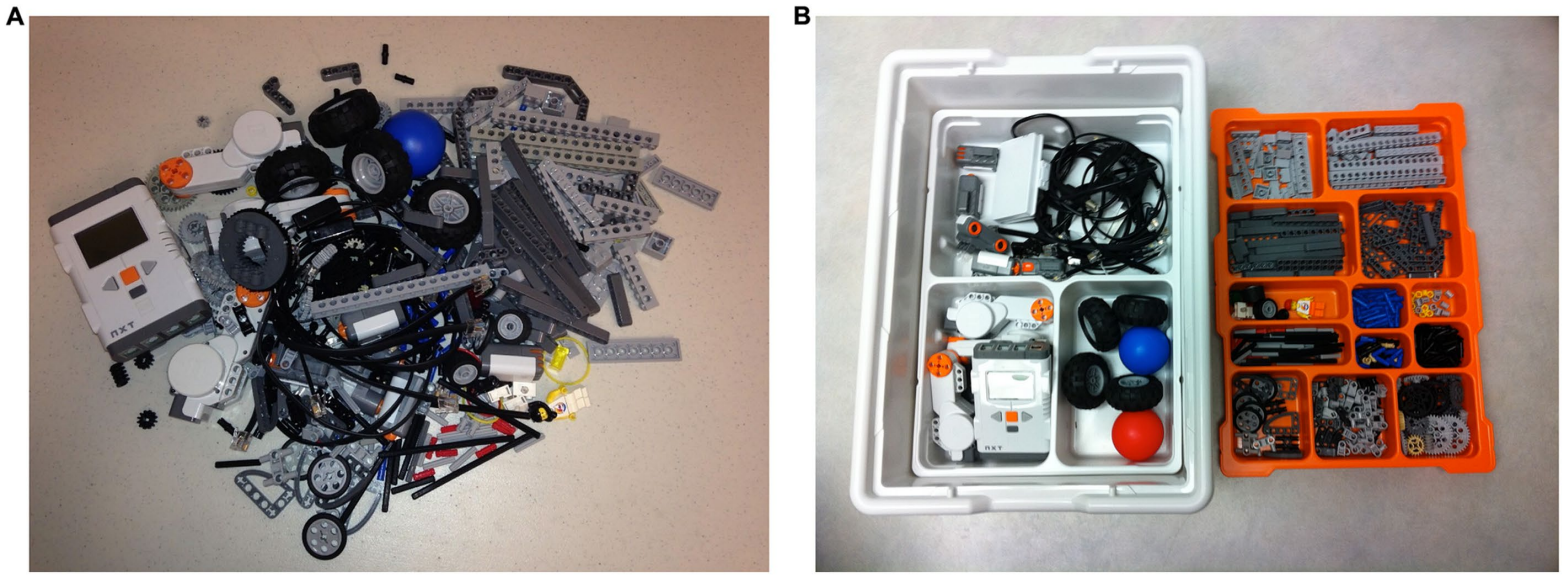
Task materials. **(A)** Shows sample mixed up Lego NXT kit. **(B)** Shows reference picture for a sorted kit.

Participants received a sample mixed up Lego NXT kit with trays (see Figure 2A), and a printed reference picture (see Figure 2B). Participants were free to manipulate Lego pieces.

#### 2.1.5 Set up

**Figure 3:**
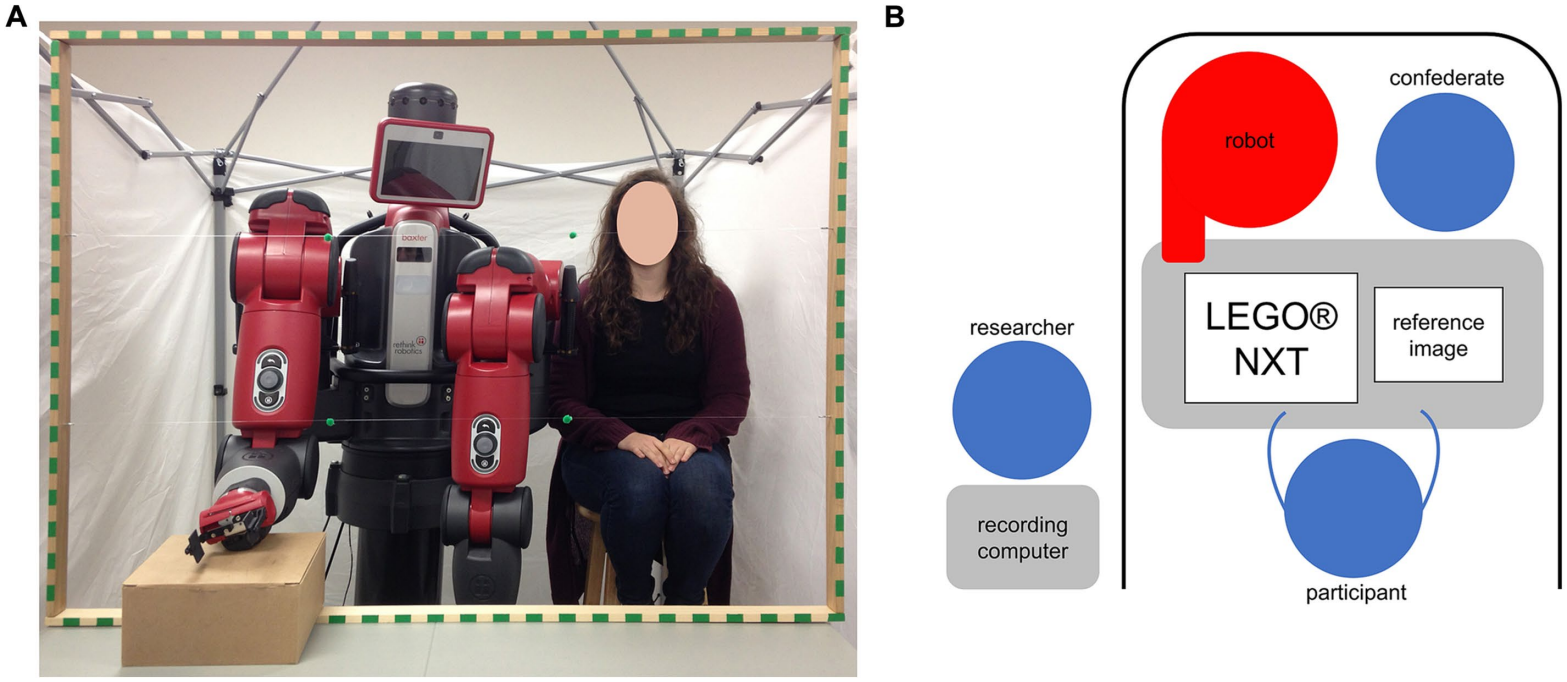
Experiment setup. **(A)** Shows participants’ view in team condition, with the wooden frame surrounding the view. **(B)** Shows a schematic view of the experiment setup from above.

Testing took place in individual sessions. The participant was seated at a table, with the intended end user positioned centrally across the table: in robot and human conditions, the user was positioned centrally, whereas in the team condition both users were positioned next to each other (see Figure 3A for participant view). In both robot and team conditions, the robot was switched off, and one arm was propped up on a plain box (due to table height). In both human and team conditions, a human confederate was seated on a high stool, with hands resting in her lap.

A wooden frame with green markers was attached to the table on the edge close to the end users (see Figure 3A); it served as a reference for mapping eye movement data. For the purpose of maintaining a clean task environment (in which only task materials, the end user, and the table were presented), the setup was surrounded on three sides by a white room divider, with the supervising researcher seated nearby but out of participant’s sight (see Figure 3B).

#### 2.1.6 Procedure

The experiment started with an introduction, i.e., the researcher explained the purpose and content of the study, followed by obtaining written consent from the participant. Shortly after, the researcher gave a short demonstration of the mobile eye tracking system to familiarize the participant with the setup; the participant put on eye tracking glasses and was instructed to move around to get comfortable with the equipment.

For eye-tracker calibration, a whiteboard with pushpins was propped against the wooden frame on the participant station to cover the entire front field of the participant’s view (positioned at arm’s length to the participant), with pushpins serving as calibration points. A three-point calibration was performed. Following initial calibration, the participant again looked at calibration points as directed by the researcher to confirm calibration accuracy; if the estimated gaze point did not overlap with calibration points at this stage, calibration was repeated until satisfactory accuracy was achieved. After calibration, the whiteboard was removed, and task materials (Lego kit and reference image) were placed on the table in front of the participant. Furthermore, the participant received a think aloud training following procedure from Tenbrink and Taylor (2015) prior to the design task.

**Figure 4:**
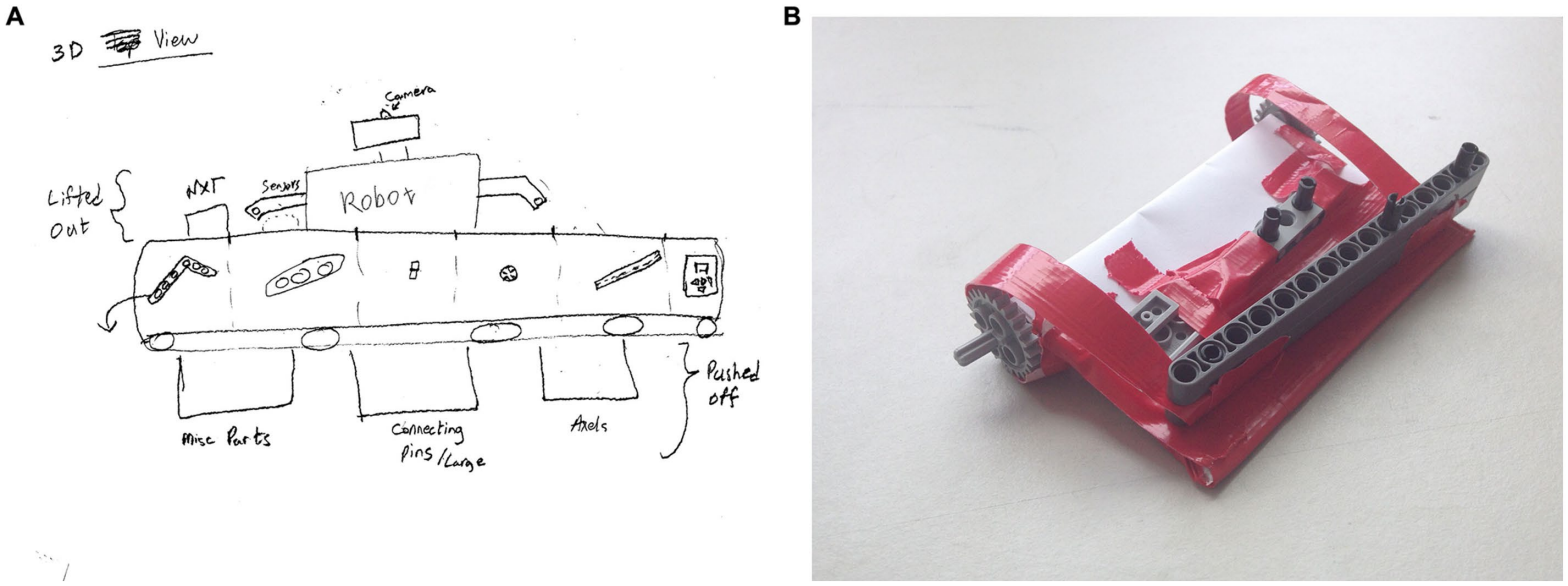
Examples for solutions provided by participants. **(A)** Shows a sketch of the tool idea drawn by a participant who worked with the robot end user. **(B)** Shows a prototype of the tool idea built by a participant who worked with the robot end user.

To begin with the task, the participant was provided with an explanation of the design problem. Specifically, the participant was instructed to conceptualize a tool (a physical piece of equipment) to optimize the sorting of Lego bricks for a particular end user (sorting must match the reference image Figure 2B). Participant might not be familiar with the full extent of robot’s/person’s capabilities. If a particular capability or feature was crucial for participant’s design, for example the height of the robot/person, the participant could make an assumption. Upon understanding the design problem, the participant was given a 10 min brainstorming time window during which to explore possible solution paths. During brainstorming, the participant could look at the end user/reference image and manipulate the Lego bricks. After the 10 min brainstorming, the participant received plain paper, pens, scissors, and tape for use in visualizing his/her tool design idea (see Figure 4 for two examples of solutions provided by participants).

### 2.2 Data set

Throughout the course of 10 min brainstorming, both think aloud and eye tracking data were simultaneously recorded, leading to a true data fusion that allowed us to investigate the cognitive processes underlying design planning. It should be noted that, in Kaszowska (2019), the intended end user for the tool (robot; human; or a team consisting of robot and human) did not affect the tool planning process. As our primary interest was to measure whether the eye movement behavior would be affected by a shift in conceptual direction, the data pre-processing and analysis presented in this work did not consider the end user factor.

#### 2.2.1 Pivot events in think aloud

The analysis of verbalizations began with a verbatim transcription and proofreading (by two researchers), during which the speech is converted from the audio recording into a written form. Importantly, each word in the transcript was automatically timestamped using Google’s Speech-to-Text Conversion (version 1) and subsequently proofread by a researcher to ensure accuracy to 100 ms.

The think aloud data analysis was based on Cognitive Discourse Analysis (Tenbrink, 2014), emphasizing two approaches to the verbal data: content-based inspection (providing in-depth insights about the designer’s current focus during solution planning), and analysis of linguistic features (reflecting patterns associated with particular concepts emergent in the protocol). Such semantic-content analysis of think aloud data enabled the identification of pivot events (instances where the verbalization indicates a shift in conceptual direction). The process of identifying pivot events involved three individuals: two coders, C1 and C2, and a referee designated as R1. The procedure unfolded through the following sequential steps: (a) Development of the pivot coding scheme: C1, C2, and R1 independently read the same three transcripts. C1 and C2 marked what they considered to be pivot events. Then C1, C2, and R1 compared their markings, addressing discrepancies through discussion. All discrepancies were resolved through discussion. In cases where C1 and C2 could not arrive at a compromise, R1 made the final decision. Based on this discussion, the final definition of pivot events was formalized. (b) C1 and C2 independently coded three new transcripts without consultation. The alpha assessing of inter-rater reliability was 0.89, exceeding the threshold of 0.8 suggested by Krippendorff (2004). (c) C1 and C2 divided the remaining transcripts between themselves and conducted independent coding. In cases of uncertainty, R1 was consulted by the individual coders.

As outlined earlier in the introduction, a pivot event is often reflected as hesitation marker—such as “hm” or “um” in verbal reporting—indicating hesitation, uncertainty, or rethinking of an idea, denoting a shift in conceptual direction during the planning of solution paths. However, it is possible in the context of concurrent think aloud that not every hesitation corresponds to a conceptual direction shift, and not every conceptual direction shift is accompanied by a hesitation marker. Nonetheless, the pivot events within think aloud transcripts were identified based on their context, that is, based on the whether the sematic content of think aloud indicated a design direction shift.

#### 2.2.2 Pivot events in eye tracking data

Eye tracking and think aloud data were matched based on event timestamps at the level of individual fixations and words. Each pivot event was identified within the timestamped transcripts. Pivot events can encompass silences and nonverbal content such as *um*, therefore the beginning and ending timestamps of pivot events were determined with regard to the surrounding timestamped words within the three-stage pivot sequence. For instance, a pivot event began at the ending timestamp of the preceding word (i.e., the last word in the trigger phrase when the participant encountered a troubling issue) and ended at the starting timestamp of the first word in the subsequent utterance (i.e., the first word in the resolution phrase). The corresponding timestamps from the transcripts were then used to identify time windows of interest related to pivot events within the eye tracking data. Fixations that coincided with the beginning and ending timestamps of each pivot event were annotated.

#### 2.2.3 Eye movements pre-processing

Pre-processing of raw eye tracking samples was performed using SMI BeGaze (version 3.5, SensoMotoric Instruments, Inc.). Fixation data was provided in the form of a text file with start/end timestamps, duration, *x*-and *y*-coordinates for fixation position, and pupil-related metrics. Saccade amplitude was determined as the distance between two fixation locations (with x and y coordinates). Furthermore, fixations that contained blinks were excluded prior to data analysis. Fixations lasting less than 100 ms were removed if they occurred on either side of a blink. Fixation durations were trimmed to remove all fixations that were less than the sampling interval of 33 ms. This removed 0.2% of the eye movement data.

### 2.3 Data analysis

All analyses of eye movements reported here were carried out using MATLAB R2022a and R statistical software (R Core Team, 2022). Fixations and saccades were analyzed in terms of fixation durations (ms) and saccade amplitudes (deg). Specifically, we examined the indications of ambient and focal modes of processing (reflected in temporal changes in fixation durations and saccade amplitudes) during the periods around the pivot events identified within the 10 min planning process. Throughout this 10 min planning process, a total of 2,854 pivot events were identified across all participants, with variations observed among participants (ranging from a minimum of 28 to a maximum of 127 pivot events). The median duration of pivot events is 1700 ms, with an interquartile range of 3,000 ms. Eye movements that occurred within this 10 min planning process constitute the database (a total of 141,958 fixations and saccades) for further processes.

As our goal was to examine whether pivot events are accompanied by transitions between ambient and focal visual processing, fixation durations and saccade amplitudes around pivot events were compared to fixation durations and saccade amplitudes during periods without pivot events (control analysis). The approach proceeds as follows (see red frame in Figure 1 for a visualization of the procedure). First, we selected 5 fixations preceding and 5 fixations following one pivot event to measure the temporal changes in gaze behavior during the periods around pivot events. The amplitude of subsequent saccades of these selected fixations were also subjected to analysis. To prevent any overlap between the selected fixations/saccades, if the interval between two pivot events was shorter than 10 fixations (e.g., when there were only 8 fixations between one pivot event end and the next pivot event start), the related metrics for both pivot events (i.e., the fixations/saccades within this interval) were excluded from the analysis. Then, to provide a comparable control analysis, we randomly selected continuous sequences of 10 fixations and their subsequent saccades from the database that did not include any pivot events (i.e., when participants were fluently verbalizing their thoughts without altering their conceptual direction) nor the pivot-related metrics (i.e., the 5 fixations/saccades preceding or following pivot events). The above procedure resulted in a total of 30,880 fixations and saccades as the dataset for pivot condition (about 34 sequences per participant, *SD* = 8.75) and a total of 39,380 fixations and saccades as the dataset for control analysis (about 44 sequences per participant, *SD* = 18.85), respectively. It is noteworthy that the utilization of an analysis window of 5 fixations before/after one pivot event enabled a more extensive analysis, covering 54% of all pivot events. In the given context, opting for a smaller analysis window allows for the inclusion of a greater number of pivot events for analysis (with consideration of the ‘no-overlapping’ selection criterion described above). Conversely, extending the analysis window to, for instance, 6 or 7 fixations before/after a pivot, would substantially reduce the number of pivot events available for analysis, covering 48 and 41% of pivot events, respectively.

**Figure 5:**
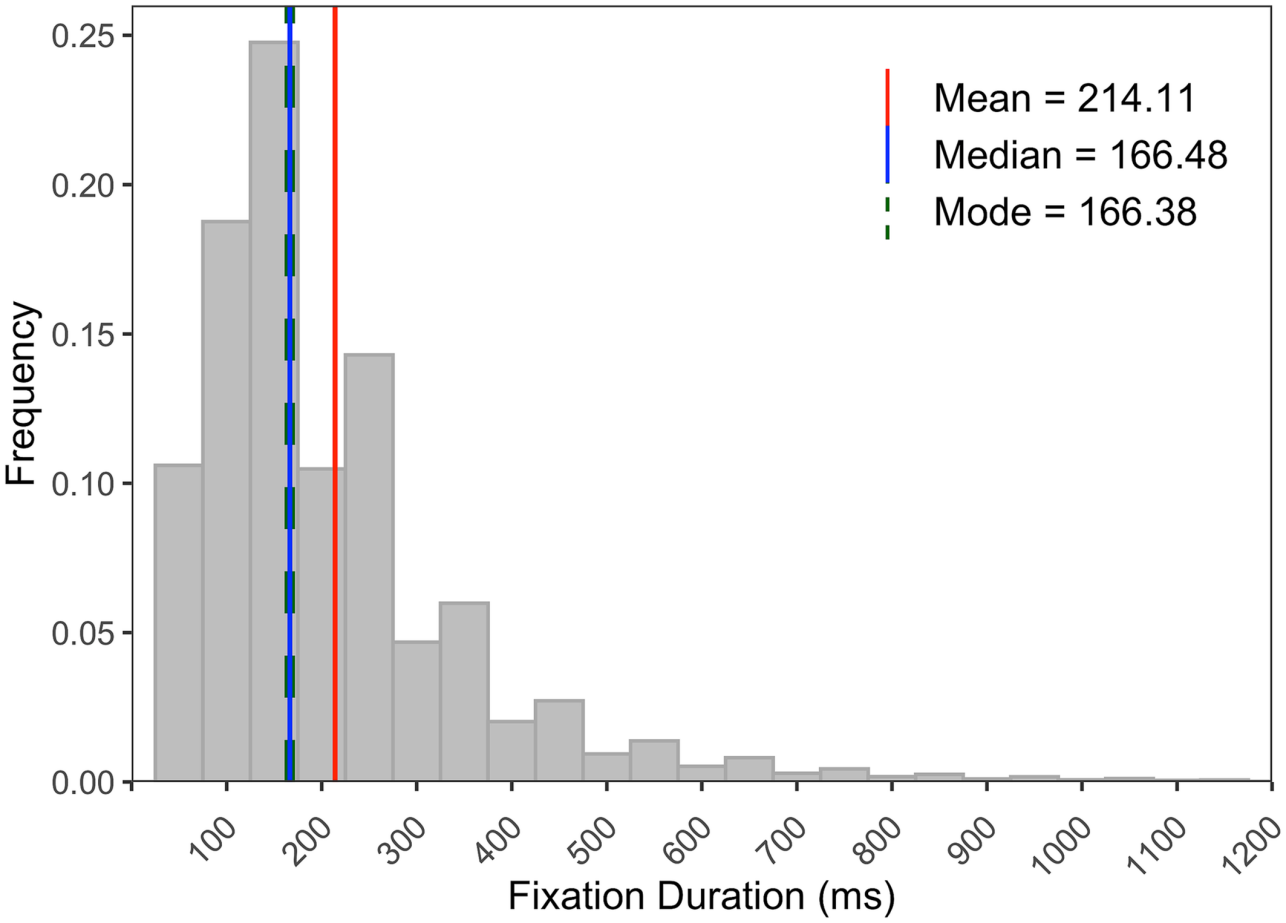
The frequency distribution of fixation durations (70,979 counts) from the database. Red, blue, and green lines represent mean, median, and mode values, respectively.

Prior to analyses of the above-created datasets, we assessed the overall distribution of fixation durations from the database. As shown in Figure 5, the distribution of fixation durations was positively skewed (skewness = 3.59) with the mode below the mean, where the median is more reflective of central tendency than the mean. Furthermore, given the likelihood of individuals exhibiting varying gaze behavior patterns around pivot events throughout the problem-solving process and the differing frequency of pivot events among participants, we aggregated data using median values, which are less affected by extreme values. Specifically, median values of fixation durations and saccade amplitudes were computed for each participant at every fixation number (corresponding to the numbering of fixations 1–10 selected around a pivot event, as depicted in Figure 1). These computed median values served as the basis for subsequent statistical analyses, including repeated measures multivariate analysis of variance (rm-MANOVA). Tests based on the MANOVA approach are free from sphericity assumptions and therefore do not lead to an inflated Type 1 error rate, which result in the more powerful test statistic if there is a contrast among the means that is reliable (O'Brien and Kaiser, 1985; Algina and Keselman, 1997; Park et al., 2009). In the rm-MANOVA, Pillai test statistic was used and reported (O'Brien and Kaiser, 1985).

## 3 Results

Before delving deeper into individual processing mechanisms, we first evaluated whether the presence or absence of pivot events might lead to distinct gaze behavior. For this purpose, the analysis window containing 10 fixations was evenly divided into two phases: (1–5 represent the phase before and 6–10 represent the phase after a pivot event); average fixation durations during these two phases (averaging the medians) were computed individually for each participant in both the pivot and control analysis. A paired *t*-test was subsequently performed to determine whether the pivot and the control analysis differed in terms of temporal changes in fixation durations. These temporal changes, specific to each participant, were calculated as the difference between the average fixation duration in the phase before and the phase after, separately for both the pivot and control analysis. The results indicated a significant difference between the pivot and the control analysis for temporal changes in fixation duration, *t*(44) = 2.15, *p* < 0.05; the mean difference between the two conditions was 7.20 ms, 95% CI [0.44, 13.96].

**Figure 6:**
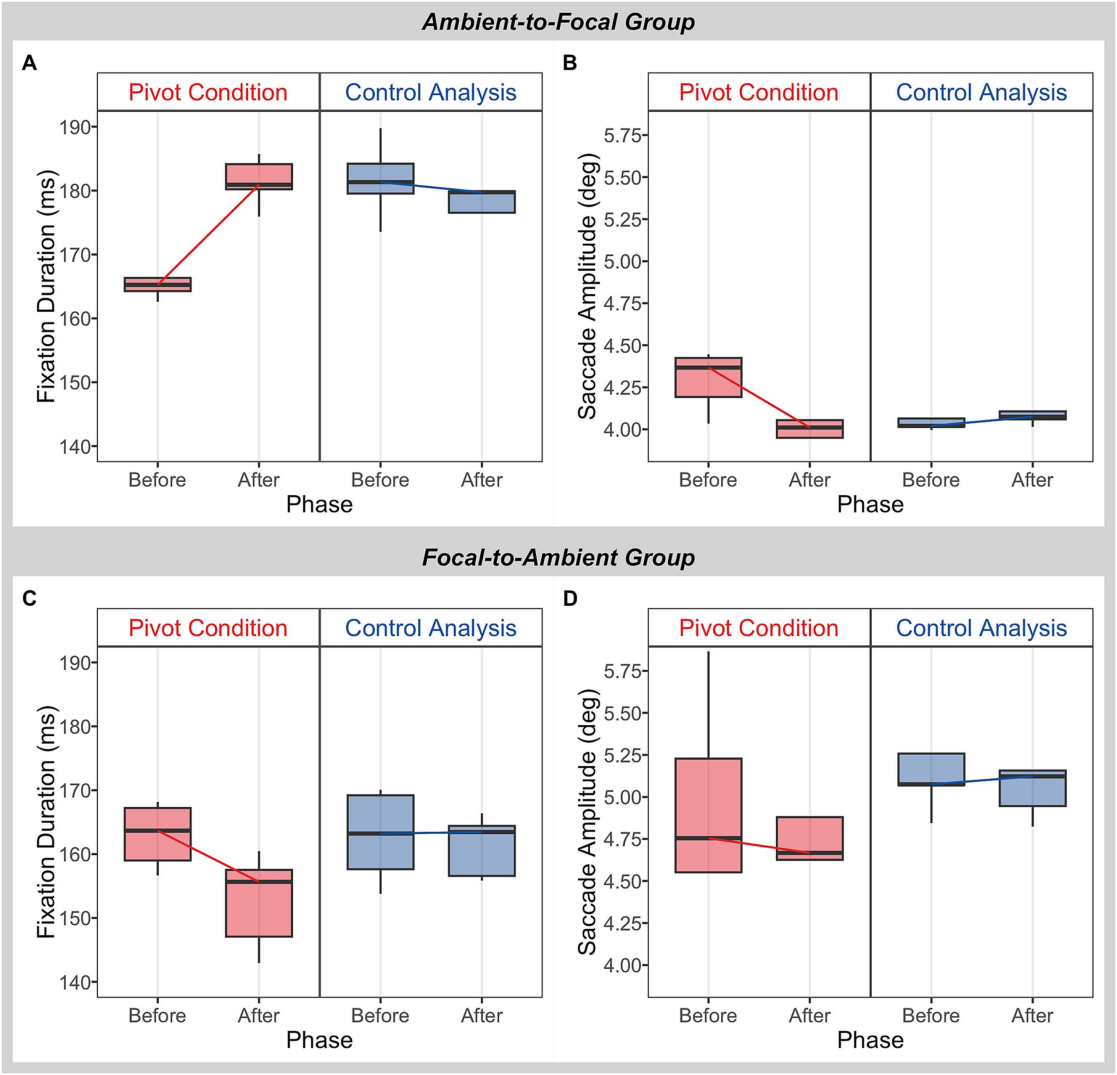
Observations of fixation durations and saccade amplitudes with and without pivot events for respective groups. Estimated marginal means (estimated under rm-MANOVAs) for fixation durations **(A,C)** and saccades amplitudes **(B,D)** are grouped into two phases based on fixation number (fixation/saccade 1–5 represent the phase before and fixation/saccade 6–10 represent the phase after a pivot event), with the box representing medians, 25 and 75% quartiles and the whiskers the range.

In order to investigate the effect of a pivot event on a finer grained level, e.g., what attentional strategy was deployed or preferred during this timeframe, we categorized participants into two groups—ambient-to-focal vs. focal-to-ambient—based on their temporal changes in fixation durations around pivot events in the pivot condition: 62% (*N* = 28) participants exhibited an increase in their average fixation durations from the phase before to the phase after pivot events, which constitutes the ambient-to-focal group; 38% (*N* = 17) participants exhibited a decrease in their average fixation durations from the phase before to the phase after pivot events, which constitutes the focal-to-ambient group. These distinct temporal changes observed for individual participant provided an initial assessment of their primary attentional strategy. For each group, custom contrast analysis within rm-MANOVA was used to validate the presence of their respective attentional strategy. Specifically, we tested two sets of contrasts: comparisons of phases (before vs. after) in each condition were performed to determine the characteristics of ambient and focal mode of processing; and comparisons of temporal changes in fixation durations/saccade amplitudes between the two conditions (pivot vs. control analysis) were performed to determine whether ambient-to-focal/focal-to-ambient processing occurs in tandem with pivot events. To set up contrasts, rm-MANOVAs were firstly conducted: rm-MANOVAs were conducted on the median values of fixation duration and saccade amplitude with the fixation number (from 1 to 10) and condition (pivot vs. control analysis), both serving as within-subjects factors, respectively (results of rm-MANOVAs see Table 1). The estimated marginal means for fixation durations/saccade amplitudes resulting from rm-MANOVAs are displayed in Figure 6. For the ambient-to-focal group (Figures 6A,B), in the pivot condition, fixation durations were relatively short and the amplitude of subsequent saccades were long during the phase before pivot events. While during the phase after pivot events, fixation durations became longer and the amplitude of subsequent saccades became shorter. For the focal-to-ambient group (Figures 6C,D), in the pivot condition, fixation durations showed a decrease from the phase before to the phase after pivot events, while the amplitude of subsequent saccades remained relatively stable with a slight decrease. On the other hand, in the control analysis, both groups displayed generally steady fixation durations and saccade amplitudes between the two phases.

**Table 1:** Results of the repeated measure-MANOVA tests on median fixation durations/saccade amplitudes for respective groups.

Group	Effect	df	*F*	*p*	Pillai’s trace
Ambient-to-Focal					
	Fixation durations				
	Fixation Number	9, 19	3.63	< 0.01	0.63
	Condition	1, 27	3.78	0.062	0.12
	Fixation Number × Condition	9, 19	5.45	< 0.001	0.72
	Saccade amplitudes				
	Fixation Number	9, 19	1.43	0.245	0.40
	Condition	1, 27	0.08	0.784	0.003
	Fixation Number × Condition	9, 19	1.69	0.161	0.44
Focal-to-Ambient					
	Fixation Durations				
	Fixation Number	9, 8	2.30	0.127	0.72
	Condition	1, 16	3.44	0.082	0.18
	Fixation Number × Condition	9, 8	0.82	0.617	0.48
	Saccade Amplitudes				
	Fixation Number	9, 8	1.83	0.203	0.67
	Condition	1, 16	0.53	0.477	0.03
	Fixation Number × Condition	9, 8	0.50	0.836	0.36

For the respective group, results of the two rm-MANOVAs (using Pillai’s trace) with condition (pivot vs. control analysis), fixation number (from 1 to 10, 1–5 represent the phase before and 6–10 represent the phase after a pivot event) as the factors for both fixation durations and saccade amplitudes.

Subsequently, custom contrasts were performed separately for the respective group based on the estimated marginal means for fixation durations/saccade amplitudes of the pivot and of the control analysis (results of custom contrasts see Table 2). The results for the ambient-to-focal group revealed significant differences in temporal changes in eye movement behavior between the two conditions: fixation durations exhibited a significantly greater increase while saccade amplitudes showed a significantly greater decrease in the pivot condition than in the control analysis. More importantly, the estimated differences (for ambient-to-focal group in Table 2) demonstrated a similar pattern to the distribution of the marginal means (see Figures 6A,B): in the pivot condition, fixation durations significantly increased from the phase before to the phase after pivot events, while saccade amplitudes showed a decrease. Nevertheless, in the control analysis, eye movement behavior exhibited a generally smaller change, namely a decrease in fixation durations and a minimal increase in saccade amplitudes. On the other hand, the results for the focal-to-ambient group showed a significant difference in temporal changes in fixation durations between the two conditions: fixation durations displayed a significantly greater decrease in the pivot condition than in the control analysis (*cf.* Figures 6C,D). No significant difference was observed between the two conditions for temporal changes in saccade amplitudes. In the control analysis, eye movement behavior remained relatively stable.

**Table 2:** Custom contrasts within repeated measure-MANOVA tests for respective groups.

Group	Contrast	Estimated difference	SE	df	t.ratio	p
Ambient-to-Focal						
	Fixation durations					
	Pivot Condition: After – Before	13.74	1.91	27	7.19	<0.0001
	Control Analysis: After – Before	−3.15	3.14	27	−1.00	0.324
	Temporal Change: Pivot Condition – Control Analysis	16.89	3.87	27	4.369	<0.001
	Saccade amplitudes					
	Pivot Condition: After – Before	−0.28	0.13	27	−2.15	0.081
	Control Analysis: After – Before	0.06	0.07	27	0.84	0.407
	Temporal Change: Pivot Condition – Control Analysis	−0.33	0.14	27	−2.36	<0.05
Focal-to-Ambient						
	Fixation durations					
	Pivot Condition: After – Before	−10.21	1.99	16	−5.13	<0.001
	Control Analysis: After – Before	−1.45	3.69	16	−0.39	0.701
	Temporal Change: Pivot Condition – Control Analysis	−8.76	3.85	16	−2.28	<0.05
	Saccade amplitudes					
	Pivot Condition: After – Before	−0.29	0.33	16	−0.87	0.790
	Control Analysis: After – Before	0.02	0.15	16	0.13	0.900
	Temporal Change: Pivot Condition – Control Analysis	−0.31	0.38	16	−0.81	0.428

For the respective group, results of the two sets of contrasts for both fixation durations and saccade amplitudes: comparisons of phase (before vs. after) in each condition; and comparison of temporal changes in fixation durations/saccade amplitudes between the two conditions (pivot vs. control analysis). SE represent standard error. Two-tailed *p*-values were corrected using Holm method.

## 4 Supplementary analysis

Past research has shown that eye movements are not solely the result of current visual processing needs, but also depend on internal (mental) representations (Richardson and Spivey, 2000; Spivey and Geng, 2001; Altmann, 2004; Ehrlichman et al., 2007). For instance, Richardson and Spivey (2000) reported eye movements to relevant regions triggered by questions about auditory and semantic—rather than visual—information acquired even when the actual stimulus/event is no longer presented. As outlined earlier, a pivot event typically occurred when participants started to be faced with a difficulty and had to choose between possible ideas/approaches to update the problem state, as a result of greater cognitive demand in language production (Barr, 2001). Suppose the onset of a pivot event reflects an impair in both the straight cognitive aspects of speech planning and the metacognitive aspects of problem-solving (in this context, participants began to shift their conceptual direction), which introduces a top-down interference to the attentional system, requiring the individual to find a new balance between internal processing versus perceptual processing. This balance shift might result in a breakdown of the “normal” moment-to-moment gaze control process and manifest itself in gaze behavior. As such, expecting a smooth transition between ambient and focal gaze behavior (e.g., fixation durations steadily increase while saccade amplitudes decrease) at this very moment may be somewhat oversimplified. Correspondingly, an exploratory analysis of visual fixations around pivot event onset is carried out in order to investigate how people’s gaze behavior may be affected when they experience the beginning of a conceptual direction shift.

### 4.1 Data analysis

For the exploratory analysis of potential oculomotor responses to the beginning of a pivot event, we used a similar approach (see blue frame in Figure 1 for a visualization of the procedure). We selected the onset of the pivot event as a reference point and analyzed fixations occurring around this time. In particular, we defined an analysis window consisting of a continuous sequence of 9 fixations. These 9 fixations were selected as the 4 fixations preceding and the 4 fixations following the one during which the pivot event began. Pivot events were excluded from analysis if they spanned fewer than 5 fixations or if the interval between them was shorter than 4 fixations (for the purpose of ensuring the 4 fixations preceding the pivot event onset did not overlap with the end of previous pivot event). In order to prevent any potential influence of ambient/focal processing occurring after pivot events on our observations, the 4 fixations following the pivot event onset should take place within the pivot event itself. This procedure resulted in 11,394 fixations as the dataset for further analysis (about 28 sequences per participant, *SD* = 7.01).

Moreover, as we suppose that visual fixations might work as an instantaneous indicator of the onset of a pivot event, an assessment of how different fixations are distributed around the time of pivot event onset was also of interest. This approach is beneficial for localizing effects in time since it can show whether the predicted behavior (e.g., a change in fixation duration) is primarily attributable to the pivot event onset. For this purpose, the above analysis was extended with two additional approaches. First, we categorized fixation durations (11,394 fixations forming the above dataset) into tertiles (i.e., short, intermediate, and long) and compared their frequencies around the onset of the pivot event. Second, we evaluated fixation durations prior to the occurrence of any pivot event. Specifically, we defined an observation period for each participant that started with the think aloud onset and terminated before the occurrence of the first pivot event (for an example, see the lower part of the blue frame in Figure 1). The duration of this observation period as well as the median value of the fixation durations within it (about 42 fixations per participant) were then computed for each participant individually.

### 4.2 Visual fixations as an instantaneous indicator of pivot event onset

**Figure 7:**
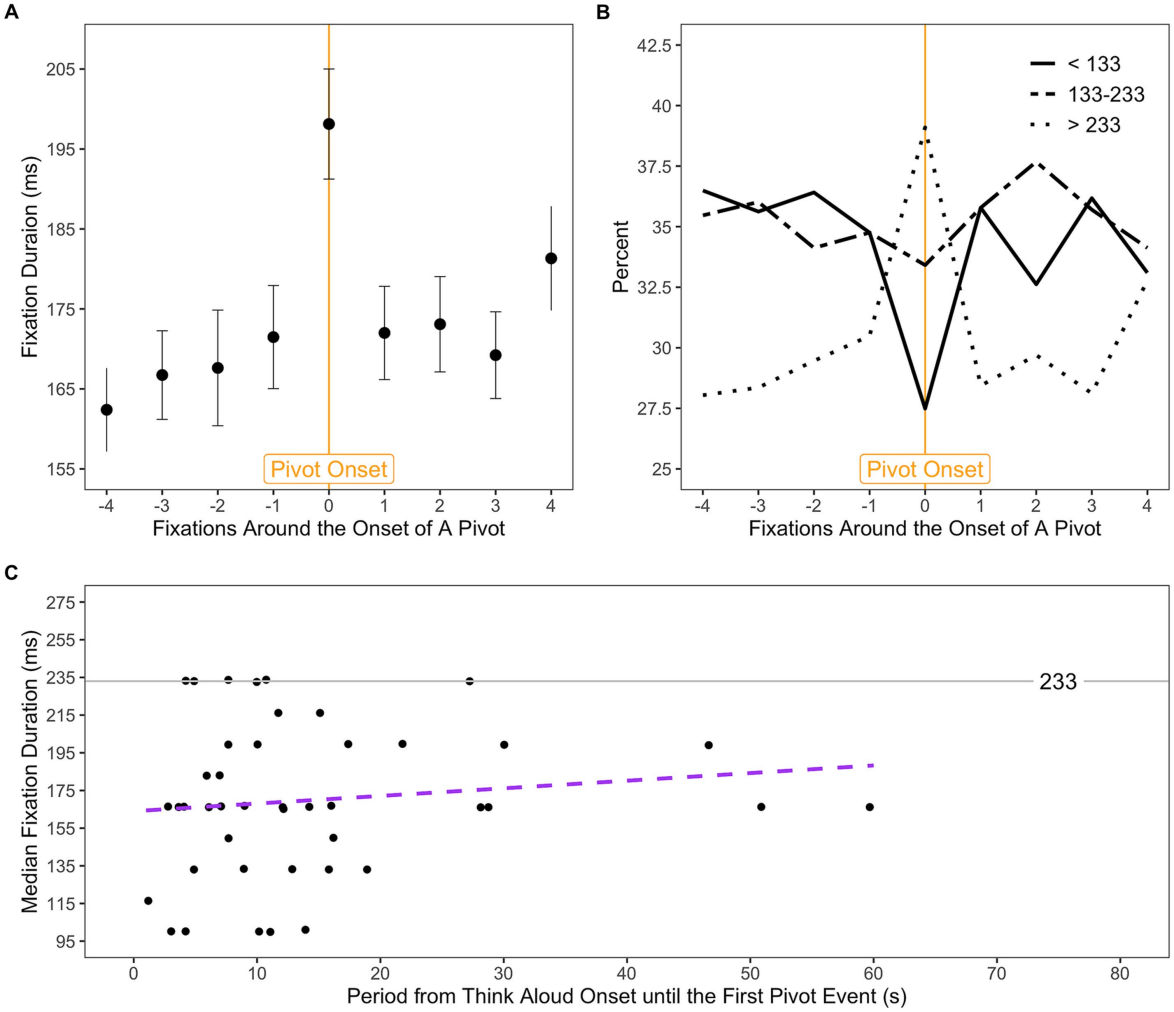
Visual fixations relative to the onset of the pivot event. Estimated marginal means (estimated under rm-MANOVA) for fixation durations are plotted over the fixation number relative to the onset of a pivot event **(A)**. The “0” corresponds to the fixation at the moment of the pivot event began. Error bars represent ±1 standard error. Frequencies of fixation durations of three categories around the onset of a pivot event **(B)**. Median fixation duration within the period ranging from think aloud onset until the first pivot event as a function of the period duration is plotted as data point for each individual participant **(C)**, with two outliers (data points located on the x-axis above 100 s) excluded. The purple dashed line represents linear regression fit to the data.

To investigate the effect of the beginning of a pivot event on visual fixations, an rm-MANOVA was conducted on the median values of fixation durations (computed for each participant at every fixation number, ranging from “-4” to “4” relative to the onset of the pivot event, as depicted in Figure 1), with the fixation number serving as within-subjects factor. Analysis revealed significant changes of fixation durations over the fixation numbers, *F*(8, 37) = 5.06, *p* < 0.001, Pillai’s trace = 0.52. The estimated marginal means for fixation durations resulting from the rm-MANOVA are displayed in Figure 7A. As shown in Figure 7A, an instantaneous prolongation of fixation can be observed at the moment of the pivot event onset (numbered as “0”). Interestingly, the durations of the fixations preceding and following the pivot event onset do not seem to be affected much by the occurrence of pivot event. The follow-up *post hoc* pairwise *t*-test comparisons (Holm corrected) confirmed that the fixations during which the pivot event began were significantly longer than fixations both preceding and following the pivot event onset (all *p* < 0.05), with the exception of the latest fixation within the pivot event numbered as “4” in Figure 7A. No significant difference was found between fixations preceding and following the pivot onset.

Another interesting effect can be seen in Figure 7B, as we compared frequencies of the three categories of fixation durations (categorized into tertiles) around the onset of the pivot event. It clearly demonstrates that the three categories of fixation durations were affected by the beginning of a pivot event in different ways: there was a considerable reduction of relatively short fixations (category less than 133 ms) and an increase of longer fixations (category above 233 ms). Finally, the assessment of fixation durations within the period ranging from think aloud onset until the first pivot event (see Figure 7C) revealed that, for instance, participants tended to conduct relatively short fixations (e.g., shorter than 233 ms) until they approached the first pivot event. Meanwhile, the linear regression slope in Figure 7C shows a positive correlation between fixation duration and the period from think aloud onset until the first pivot event, indicating that when it took longer for participants to reach the first pivot, longer fixations tended to be involved. However, this correlation exceeded statistical significance (*r* = 0.404, *p* = 0.411).

## 5 Discussion

Solving problems in real-world settings is an inherently complex process that requires people to systematically evaluate and update the problem states which lie on different possible solution paths linking the initial problem state and the goal state (Newell and Simon, 1972; Kounios et al., 2008; Barbey and Barsalou, 2009). The work presented here investigated eye movement behavior when participants explored possible solution paths to a complex tool design problem, and gathered additional insights into the relationship between visual attention and internal thinking underlying solving processes. In particular, we analyzed participants’ eye movements within and around the pivot events (i.e., the specific instances where participants shifted the conceptual direction as they planned solutions to the design problem) identified within the concurrent think aloud protocol (participants verbalized ongoing thought processes out loud). The analysis of eye movements around pivot events suggested a deployment of the ambient-to-focal rather than focal-to-ambient visual processing—participants’ fixation durations increased while saccade amplitudes decreased from the periods preceding to the periods following pivot events. In contrast, such characteristics were not observed in the control analysis which did not involve pivot events, indicating that the ambient-to-focal processing occurred in tandem with shifts in conceptual direction during problem-solving. Furthermore, the analysis of visual fixations around the pivot event onset revealed a significant increase in fixation duration at the moment of the pivot event onset, suggesting that starting a shift in conceptual direction manifests itself in oculomotor behavior.

### 5.1 Deployment of ambient and focal attention in problem-solving

Viewing change of problem representation as a search process for solution allows us to account for complex problem-solving (Newell and Simon, 1972; Kaplan and Simon, 1990; Ohlsson, 1992), such as tool designing. In light of the crucial role that perceptual processes play in generating problem representations (Simon, 1978), it is possible that when solvers encounter difficulties compelling them to shift their conceptual direction in order to adjust the solution path, they employ certain perceptual strategies to facilitate representing critical features of the task environment in their conceptual representation of the problem. We therefore hypothesized that a switch between ambient and focal visual attention would be useful during that timeframe. Individuals might be differentially susceptible to this particular phenomenon. To be specific, individuals inclined toward the ambient-to-focal attentional strategy may find ambient exploratory processing enables a rapid influx of information necessary for adaptive updating of problem representation (as they are approaching the shift in conceptual direction), whereas more memory-based focal processing (as its underlying ventral stream utilizes stored representations of the information/features) facilitates a deep understanding and fluent processing of the semantic meanings of the new solution arising in mind. In contrast, individuals inclined toward the focal-to-ambient attentional strategy may find ambient processing to be an effective alternative for refining the new solution represented in the conceptual system with immediate information/features since the focal processing of the initial problem representation has reached an impasse.

Our data provide support for this hypothesis. First, distinct fixation behaviors were observed (regarding temporal changes) between the pivot condition and the control analysis without pivot events, highlighting the close interaction between perceptual and cognitive processes. Second, a more detailed analysis of eye movement behavior for participants in ambient-to-focal and focal-to-ambient groups (participants were classified based on their fixation behavior) yielded different results. In the ambient-to-focal group, participants’ fixation durations were relatively short and saccade amplitudes were long prior to pivot events, i.e., where participants approached the shift in conceptual direction, suggesting ambient mode of processing. Once pivot events were over (the conceptual direction has shifted), fixation durations became longer and saccade amplitudes became shorter, suggesting focal mode of processing. Alternatively, one could only accept that the main rm-MANOVA analysis shows saccade amplitudes interacting with the control analysis for the ambient-to-focal group (refer to the interaction between two factors in Table 1), but this is not necessarily the case in the given context. Over a pivot sequence where the participant sustained focus on a particular object/event (an instance where the troubling trigger and resolution phrases fall within the same content category), larger saccades for ambient viewing (e.g., registering different aspects of the reference image) are not essential for a much smaller attentional window. Therefore, it is possible that the saccade amplitudes show significant differences in the contrast analysis with more nuanced time modeling that the overall rm-MANOVA did not capture. The phenomenon observed in the pivot condition is fundamentally different from the control analysis. In the control analysis, participants’ fixation durations and saccade amplitudes remained generally stable over time—fixation durations remained long and saccade amplitudes remained short—as they were fluently thinking aloud without altering their conceptual direction, demonstrating their involvement in a rather stable focal processing. The differing gaze behavior between the pivot and the control analysis may be attributed, in part or fully, to the hypothesis that the occurrence of a shift in conceptual direction drives the change from ambient to focal visual processing. Our view that ambient to focal visual attention effectively complements shifts in conceptual direction connects nicely with a view reported by Guo et al. (2022), in which ambient to focal processing behavior is found to be deployed in task processing and is closely relative to the inner mental model, i.e., mental shifts between different tasks lead to such gaze behavior.

On the other hand, the focal-to-ambient attentional strategy would reverse this pattern. However, in the focal-to-ambient group, no focal to ambient behavior was identified for saccade amplitudes, and the statistical difference between the pivot and the control analysis was not significant for saccade amplitudes. A possible explanation for the observation that fixation durations and saccade amplitudes both showed a decrease from the phase before to the phase after pivot events is that the short fixations and saccades may serve for rapid information sampling and retrieval, facilitating the refinement of the newly generated solution. After a shift in conceptual direction, the problem representation is updated with critical visual features, and a determined solution pops into mind. However, refining this updated problem representation may require returning the eyes to the critical features for retention and activation of semantic representations and identities of attend features (Henderson and Hollingworth, 2006; Ferreira et al., 2008). Given that solvers are already acquainted with the critical features embedded in their updated mental representation (e.g., their locations and spatial relations), exhaustive scanning of the task environment to obtain broad information is no longer necessary. Instead, short fixations and saccades may be more effective means of revisiting the target features, allowing for rapidly sampling and retrieval of additional aspects of information within rather specific attentional windows. It is therefore possible that this eye movement behavior is a result of strategic decisions aimed at minizine the working memory load, rather than retaining a detailed visual representation of the external world (Hayhoe and Ballard, 2005; Henderson and Hollingworth, 2006).

Furthermore, it is noteworthy that the mean saccade amplitudes for the focal-to-ambient group, in both the pivot and control analysis, are distinctly larger than those observed for the ambient-to-focal group (*cf.* Figures 6B,D). This observation underscores the existence of diverse perceptual processing behaviors employed during problem-solving, which are evident through distinct eye movement patterns, specifically in fixation-and saccade-related metrics.

Conversely, considering the reported temporal changes in fixation durations/saccade amplitudes around pivot events were relatively small, one could argue that the observed differences may be attributable to possible measurement error. This consideration is pertinent when taking into account that the data were sampled at 30 Hz, which could lead to less precise characterization of eye movement events (Anantrasirichai et al., 2016; Mack et al., 2017; Stein et al., 2021). However, it is unlikely that the differing eye movement patterns between the pivot and the control analysis were due to an effect of measurement error, especially given that the overall value of fixation durations was considerably small (see Figure 5, the mode value) and the differences in temporal changes of fixation durations and saccade amplitudes between the two conditions were statistically significant for the ambient-to-focal group. Instead, it is possible that individual differences among participants (e.g., differences in the frequency of pivot events and in fixation-and saccade-metrics, *cf.* y-axis values of fixation duration and saccade amplitude between the two groups in Figure 6), or variations in the characteristics of pivot events (e.g., duration, magnitude, and the speaker’s conceptual focus before/after a pivot), led to the predicated features of ambient and focal visual attention, but lack of a more compelling distinction. From a methodological perspective, however, eye tracking data with higher temporal resolution would be desirable in order to underpin the results presented here. Furthermore, in an extended self-paced task, inherent intraindividual differences become relevant; participants may exhibit diverse patterns of gaze behavior throughout the task, involving shifts from focal to ambient, ambient to focal, or maintaining a consistent pattern. Collecting and aggregating data from these events for each participant could have potentially reduced the statistical differences when analyzing temporal changes in fixation durations and saccade amplitudes. Future research that delves into intraindividual differences within extended complex tasks will shed light on how gaze behavior evolves over time or in response to varying events.

It is also worth noting that the time scale used in the current study for measuring ambient and focal modes of processing is vastly different from that used in earlier studies. Previous research looked at changes in attentional processing over a few seconds of time. For instance, in scene viewing studies, ambient and focal eye movement characteristics were monitored over time courses ranging from 20 fixations (Velichkovsky et al., 2005) up to 20 s (Unema et al., 2005; Eisenberg and Zacks, 2016). Other than well-controlled scene viewing tasks in which participants attend to the stimulus for a specified amount of time as instructed, Guo et al. (2022) measured ambient and focal gaze behavior over a 3 s time scale based on the results of gaze distribution analysis. However, these analysis windows are unlikely to generalize to our study, since participants were free to view and interact with the real-world task environment during the problem-solving process. It is difficult to predict the precise moment at which participants will become aware of the impasse in their solution paths. Considering that the size of the analysis window directly affects the number of pivot events included in the analysis (as detailed in the Data Analysis section), estimating 10 fixations/saccades (study phase) around the pivot event can be a more versatile approach across various conceptual shifts.

Importantly, pivot events, signifying shifts in conceptual direction, occurred frequently in the think aloud data of our study. These frequent conceptual shifts can be attributed to two factors. First, it is essential to note that not all conceptual shifts in our analysis are qualitatively equal; they vary in magnitude, involving shifts between main ideas or different perspectives on the same idea. Since our primary focus is on identifying qualitative attentional shifts in relation to shifts in conceptual direction, including all types of conceptual shifts allowed for a more extensive analysis. Second, participants were actively engaged in brainstorming, a process characterized by frequent shifts in conceptual direction as individuals explored possible avenues to solve the problem and promptly evaluated ideas. Additionally, it is noteworthy that the analysis approach for identifying pivot events in think aloud was an original contribution by Kaszowska (2019). Think-aloud studies in complex tasks generally look at broad themes explored by participants (e.g., Gagné and Smith, 1962; Van Gog et al., 2005; Ward et al., 2011; Elling et al., 2012) rather than delving into specific problem-solving strategies as approximated by minor changes in language.

Overall, the point to be emphasized here is that while different eye movement behaviors were identified around conceptual direction shift, one apparent similarity is that the control of eye movements is contingent on the specific circumstances under which differences in cognitive processes are exhibited, e.g., the presence of conceptual direction shift is accompanied by a change in gaze behavior. Moreover, the available evidence supports the formulation of the ambient-to-focal attentional strategy in problem-solving, even though it is not always employed by everyone. Nevertheless, given the considerations and limitations mentioned above, as well as the relatively subtle distinctions observed between ambient and focal eye movement characteristics, our findings should be considered provisional, and this study requires replication in another venue to test the reproducibility and consistency of our findings. Further work to bridge the gap between the laboratory analysis conclusions and the data observed in real-world settings is needed to uncover more powerful evidence of the effects of conceptual shift on ambient and focal attentional mechanisms.

### 5.2 Supplementary analysis: oculomotor response to the beginning of a conceptual shift

Our supplementary analysis is largely exploratory. Given that starting a shift in conceptual direction interrupts the ongoing thought processes during problem-solving, impairing cognitive performance (i.e., speech planning) by competing for the individual’s limited attentional resources, and thereby allowing additional information to be processed for reconstructing/updating the problem representation, it seems plausible to expect oculomotor behavior to display a notable response to the onset of a pivot event. The results provide broad support for this hypothesis. The analysis of fixation durations around the moment of pivot event onset indicates that fixation responses to the beginning of pivot events were strong, reliable, and rapid with a significant prolongation (see Figures 7A,B). More importantly, such long fixations (e.g., longer than 233 ms) seem mostly allocated to the moment of pivot event onset (see Figures 7B,C).

**Figure 8:**
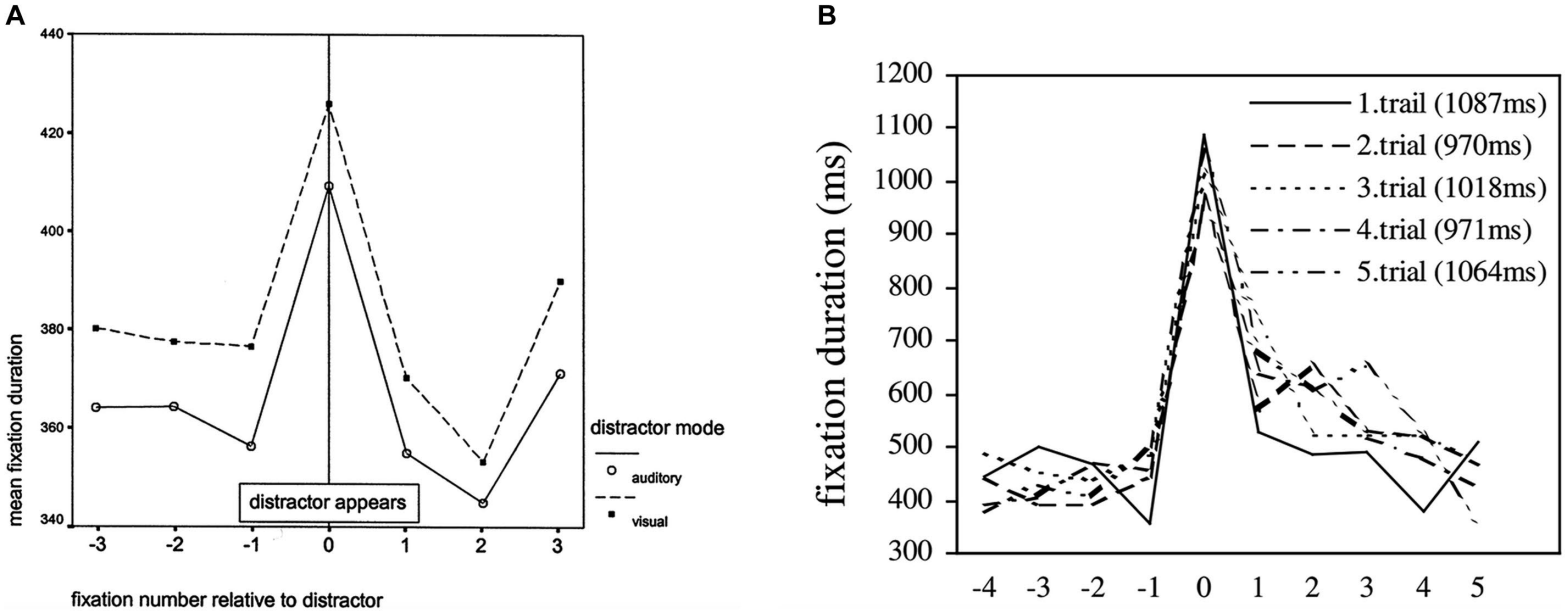
Comparable oculomotor responses from earlier studies. Panel **(A)** is adapted from Pannasch et al. (2001) with permission, depicting the presentation of a visual or auditory distractor prolonging fixations during unspaced scene viewing. Panel **(B)** is adapted from Velichkovsky et al. (2002) with permission, depicting an instantaneous increase in fixation duration when participants detected an immediate hazard in a driving simulation task.

In search of factors that may clarify the observed effect, a group of possible explanations have emerged. These explanations are not mutually exclusive and could operate in a parallel fashion. One is that the prolongation of fixations may be an expression of the orienting response (Pannasch et al., 2001; Graupner et al., 2007). Prior findings demonstrating that the presentation of a distractor in stimulation (Graupner et al., 2007; Pannasch et al., 2011; Devillez et al., 2017) or abrupt visual changes, such as the appearance of new objects (Brockmole and Henderson, 2008) or the emergence of hazard events (Velichkovsky et al., 2002), captures attention immediately and results in an effect on the duration of a current fixation. Especially, through earlier statements that can be found in a well-known paradigm of eye tracking research, namely distractor presentation experiments, a comparable behavior of visual fixation was observed for both visual and auditory distractors during scene viewing (see Figure 8A); and this observation was interpreted within the framework of novelty-based reactions such as orienting response (Pannasch et al., 2001; Graupner et al., 2007; Pannasch and Velichkovsky, 2009). The orienting response describes the behavioral and physiological responses, such as a deceleration of heart rate (Graham and Clifton, 1966) or distinctive meaningful brain network activity (Williams et al., 2000), to any novel or significant events. Such an orienting response is thought to signal the active orientation of attention towards these events and to facilitate event processing. In difference to fixation responses to meaningless distractors, similar effects were later reported in a study of hazard-related changes in fixation durations (Velichkovsky et al., 2002). Velichkovsky et al. (2002) found that during a driving simulation task, when participants detected a hazardous event (i.e., dangerous traffic situation), fixation duration remarkably increased, despite the numerous repetitions of the hazard event across 5 consecutive drive trials (see Figure 8B). The authors explained this behavior as participants engaged in more focal processing of the detailed information from the hazard event, which was considered necessary to avoid an accident.

Our finding replicated the results from the above-mentioned eye tracking studies—despite the substantial difference being that there was no novel or significant external event involved in the current experimental paradigm—a prolongation of fixations always appeared. In the present study, all changes in eye movement behavior were based on internal process changes, following closely the actual path of thinking. It is interesting to note that although internal and external events are not automatically analogous, they both manifest themselves in oculomotor behavior in the same manner. Rather than relying on external triggers, the onset of updating internal representation of a problem could also evoke a notable fixation response (i.e., instantaneously prolonged fixation duration). However, the view that an internal event could elicit orienting reflex will need to be justified by future investigations.

Another possible explanation for the finding that fixations were longer at the moment of pivot event onset is that longer fixation durations are associated with the internalization of attention (Foulsham et al., 2013; Krasich et al., 2018; Zhang et al., 2021). It seems puzzling that, as noted earlier, fixation durations are typically used to identify transitory information-processing priorities of the visual system, with longer fixations classified as focal level processing that is associated with a more in-depth analysis of visual information (*cf.* Unema et al., 2005; Pannasch et al., 2008; Fischer et al., 2013). However, studies of the relationship between mind wandering and eye movements in the contexts of scene perception (Krasich et al., 2018; Zhang et al., 2021) and reading (Reichle et al., 2010; Faber et al., 2018) demonstrate that the longer fixation duration may also indicate lesser engagement with visual information processing. For instance, mind wandering was found to be associated with fewer and longer fixation durations on the scene compared with reports of attentive viewing during real-world scene viewing (Krasich et al., 2018). Mind wandering can be viewed as a state where one’s attentional priorities shift away from external environment towards internal thoughts and feelings (Smallwood and Schooler, 2006), e.g., looking at the visual surroundings but thinking of something else irrelevant to the primary task. Experiencing conceptual direction shifts (pivot event) in problem-solving may share important information-processing characteristics with mind wandering—as it also requires the shift of attention away from online sensory information towards internal conceptual representation of the problem, which involves the explicit internalization of attention. In our case, however, internally focused attention is still intentionally directed toward the primary task, i.e., tool design. Considered in this light, when attentional resources decouple from external information and turn inward for the purpose of reorganizing/updating problem representation at the onset of a pivot event, visual processing may become less efficient. Consequently, the benefit of maintaining the current fixation may increase, leading to a prolonged fixation. Indeed, previous research has suggested that difficulties at the level of visual and cognitive processing can delay or even cancel saccade initiation, thereby extending the durations of fixations (Yang and McConkie, 2001; Nuthmann et al., 2010).

Based on this view, our data suggest that eye movements could be used to predict the moment-to-moment information-processing prioritization of the visual system across changing attentive states—from attending to ongoing task-relevant stimuli to focusing on internal representation. This finding can serve as an important springboard for future research exploring the potential uses of real-time gaze-based detection of conceptual shifts in applied context, e.g., task processing or problem-solving.

In addition to the above-discussed explanations, a more “low-level” account suggests that the observed transient increase in fixation duration may be attributable to the synchrony with the vocal response used to define the onset of the pivot event in the think aloud protocol. Sensorimotor synchronization refers to a situation in which an action is coordinated with a predictable external event, especially one characterized by periodic or rhythmic patterns (Large and Jones, 1999; Repp, 2005). This phenomenon has been extensively investigated in the context of simple repetitive tasks, where participants are required to accompany a stimulus with a simple movement, for example, finger tapping to a visual, auditory or combined auditory–visual metronome (e.g., Aschersleben, 2002; Chen et al., 2002; Repp, 2003). Interpreting the transient increase in fixation duration as a result of some form of sensorimotor synchronization would imply that the observed transient prolongation of fixation is an epiphenomenon stemming from the specific paradigm—namely, the co-registration of eye movements and a think aloud protocol—rather than being linked to problem-solving. Nevertheless, this explanation appears less plausible, given that participants were continuously speaking (thinking aloud) both before and after this specific vocal response. Future investigations are required to explore the potential influence of sensorimotor synchronization on human gaze behavior.

## 6 Conclusion

The current work investigated eye movement behavior while participants adjusted their solution paths to a complex open-ended design problem, which revealed an intimate relationship between visual attention and cognitive processes underlying problem-solving. Adding to the existing knowledge about ambient and focal visual attention, our data provide preliminary support for the existence of ambient and focal attentional processing during realistic complex problem-solving by demonstrating temporal dynamics in fixation durations and saccade amplitudes during the periods around shifts in conceptual direction. Moreover, our data demonstrate that the beginning of a shift in conceptual direction is observable in oculomotor behavior with a significant prolongation of fixations. The findings presented here show a direct relation between patterns of eye movement and modes of attentional processing, which offers a point of departure for future research into ambient and focal attentional mechanisms in higher—semantic and metacognitive—levels of information processing during complex tasks. As a result, eye tracking will become of greater importance in the development of future applications, as well as part of future applications themselves.

## Data availability statement

The raw data supporting the conclusions of this article will be made available by the authors, without undue reservation.

## Ethics statement

The studies involving humans were approved by Tufts University Social, Behavioral, and Educational IRB. The studies were conducted in accordance with the local legislation and institutional requirements. The participants provided their written informed consent to participate in this study. Written informed consent was obtained from the individual(s) for the publication of any potentially identifiable images or data included in this article.

## Author contributions

YG conceptualized the study, conducted the data analyses, drafted and revised the manuscript under supervision of SP and JH. SP, JH, and AK reviewed and approved the final version of the manuscript for submission. All authors contributed to the article and approved the submitted version.
